# Reward-related functioning across diabetes subtypes: associations with glycaemic control

**DOI:** 10.3389/fendo.2026.1959733

**Published:** 2026-09-18

**Authors:** Ayse Kurtulmuş, Fatma Büşra Parlakkaya Yıldız, Emek Esra Sahinbaş, Merve Özyıldırım, Pınar Ertunç, Zülal Çelik, Mümtaz Takır

**Affiliations:** 1 Department of Psychiatry, Faculty of Medicine, Istanbul Medeniyet University, Istanbul, Türkiye; 2 Department of Genetics, Aziz Sancar Institute of Experimental Medicine, Istanbul University, Istanbul, Türkiye; 3 Department of Endocrinology, Faculty of Medicine, Istanbul Medeniyet University, Istanbul, Türkiye

**Keywords:** anhedonia, behavioral inhibition, delay discounting, diabetes, HbA1c

## Abstract

**Background:**

Insulin signaling has been implicated in reward processing and motivational regulation through its modulatory effects on mesolimbic pathways. However, reward-related behavioral functioning across different diabetes subtypes and insulin-related metabolic states remains poorly understood. This study aimed to compare motivational and reward-related behavioral measures among individuals with type 1 diabetes mellitus (T1DM), type 2 diabetes mellitus (T2DM), prediabetes, and healthy controls, and to investigate their associations with diabetes self-management and glycaemic control.

**Methods:**

This cross-sectional study included 44 individuals with T1DM, 43 with T2DM, 28 with prediabetes, and 46 healthy controls. Participants completed measures assessing behavioral inhibition/activation (BIS/BAS), anhedonia, delay discounting, depressive symptoms, and diabetes self-management. Group differences were examined using analyses of covariance controlling for age, sex, education level, and depressive symptoms. Correlation and hierarchical regression analyses were conducted to evaluate associations with diabetes self-management and HbA1c levels.

**Results:**

Significant group differences were observed for BIS and BAS Reward Responsiveness scores after false discovery rate correction (p = .004). *Post hoc* analyses demonstrated that individuals with T1DM had significantly lower BIS and BAS Reward Responsiveness scores compared to T2DM, prediabetes, and healthy controls. No significant group differences were observed for anhedonia or delay discounting measures. Reward-related behavioral measures explained additional variance in HbA1c (ΔR² = .038, p = .035), with lower BAS Drive associated with higher HbA1c after adjustment for demographic and clinical covariates (β = −.162, p = .036).

**Conclusions:**

Altered reward responsiveness and behavioral inhibition may be particularly associated with T1DM rather than T2DM or prediabetes. Reward-related behavioral functioning also showed a modest association with glycaemic control, with BAS Drive demonstrating the most consistent individual association with HbA1c. These findings highlight the potential clinical relevance of motivational and reward-related processes in diabetes and suggest that considering these behavioural dimensions may help inform more individualized approaches to diabetes management and glycaemic control.

## 1 Introduction

Diabetes mellitus is a chronic metabolic disease characterised by impaired glucose homeostasis and systemic complications. Type 1 diabetes mellitus (T1DM), type 2 diabetes mellitus (T2DM) and prediabetes share certain metabolic abnormalities, but the insulin physiology and metabolic mechanisms of these conditions are substantially different. T1DM is characterised by an insulin deficiency due to autoimmune destruction of pancreatic β-cells. Prediabetes is generally an early hyperinsulinemic insulin-resistant state, whereas T2DM is a more advanced stage of metabolic dysregulation with persistent insulin resistance and progressive β-cell dysfunction (1, 2). In addition to its peripheral effects on glucose metabolism, there is increasing evidence that insulin has an important neuromodulatory role within the central nervous system, especially with respect to dopaminergic pathways involved in reward processing and motivational behavior (3, 4).

Insulin receptors are present throughout the mesolimbic and mesocortical dopaminergic regions, including the ventral tegmental area and the nucleus accumbens (3). Insulin has been shown experimentally to modulate dopamine transporter activity, dopamine release and reward-related signalling pathways (4, 5). Through these mechanisms, altered insulin signalling may influence reward sensitivity, reinforcement learning, motivational salience, and decision-making processes (6–8). Therefore, different metabolic states associated with insulin deficiency or insulin resistance may differentially affect reward-related behavior.

Several behavioral constructs may reflect dopamine-related motivational and reward processing. Behavioral activation and reward responsiveness are associated with approach motivation and reward sensitivity, while behavioral inhibition reflects sensitivity to punishment and avoidance-related behavior (9). Likewise, anhedonia refers to the reduced ability to experience pleasure, and delay discounting is the preference for smaller immediate rewards over larger delayed rewards and has been linked to impulsive decision-making and altered reward valuation (10). These behavioral dimensions are believed to be closely related to dopaminergic reward processing and motivational regulation (10, 11).

Motivational and reward-related processes may be particularly relevant in diabetes not only because of their etiological implications but also due to their potential contribution to disease-related outcomes. Effective diabetes management requires the long-term valuation of reward, behavioral regulation and adherence to complex self-care behaviors that require sustained motivation. Dietary regulation, physical activity, glucose monitoring, and medication adherence often necessitate prioritising delayed health benefits over immediate gratification. Therefore, alterations in reward responsiveness, behavioral inhibition, or delay discounting might be involved in difficulties in diabetes self-management and subsequent effects on glycaemic control. Previous work has mainly focused on depression, cognitive dysfunction, or general psychiatric symptoms in diabetes, while motivational and reward-related functioning in different insulin-related metabolic states is relatively underexplored (12–14). Investigating these groups together may provide a more comprehensive understanding of the relationship between differing insulin states and motivational and reward-related functioning. In addition, examining the relationships between these behavioral measures and diabetes self-management and glycaemic control may help clarify the potential clinical relevance of reward-related functioning in diabetes patients.

Therefore, the present study aimed to compare motivational and reward-related measures, including behavioral inhibition/activation, anhedonia, and delay discounting, among individuals with T1DM, T2DM, prediabetes, and healthy controls. To the best of our knowledge, this is the first study to comprehensively assess motivational and reward-related behavioral dimensions across different diabetes subtypes. Additionally, we aimed to investigate the associations between reward-related behavioral functioning, diabetes self-management behaviors, and HbA1c levels in patients.

## 2 Methods

### 2.1 Participants and procedures

Participants were recruited from the endocrinology outpatient clinics of Istanbul Medeniyet University Göztepe Prof. Dr. Süleyman Yalçın City Hospital between July 2025 and April 2026. Diagnosis of T1DM, T2DM and prediabetes was made by endocrinology specialists based on current clinical guidelines. Healthy controls were recruited from the community and relatives of hospital staff.

Individuals aged 18 years and older who were able to provide informed consent and complete self-report questionnaires were included in the study. Participants were excluded if they had severe neurological disorders, intellectual disabilities, current psychiatric disorders, substance use disorders, psychotropic use, were pregnant or lactating, or had serious medical conditions that would impact their ability to participate in the study.

After providing written informed consent, participants completed a battery of self-reported questionnaires assessing reward-related behavioral measures and diabetes self-management. Sociodemographic and clinical information was recorded, including age, sex, education level, body mass index (BMI), smoking status and HbA1c levels. The study protocol was approved by the local ethics committee (No:2025/0355) and conducted in accordance with the Declaration of Helsinki.

An *a priori* power analysis was conducted using G*Power 3.1 for group comparisons across four groups. Assuming medium-to-large effect sizes (Cohen’s f = 0.25–0.30), α = .05, and statistical power of 80%, the required total sample size was estimated to range between 126 and 180 participants. The final sample included 161 participants (T1DM = 44, T2DM = 43, Pre-diabetes=28, Healthy Controls=46). The achieved sample exceeded the estimated requirement for an effect size of f = 0.30 but fell below that required for f = 0.25. Thus, the study was adequately powered for effects toward the upper end of the assumed effect-size range, whereas more modest effects may have remained undetected.

### 2.2 Measures

#### 2.2.1 Sociodemographic data form

A structured sociodemographic and clinical data form was used to collect information on age, sex, educational level, smoking status, body mass index (BMI) and HbA1c levels. Clinical diagnoses were verified by medical records and endocrinology evaluations.

#### 2.2.2 Beck depression inventory

Depressive symptom severity was assessed using the Beck Depression Inventory (BDI), a 21-item self-report scale evaluating cognitive, emotional, vegetative, motivational, and somatic symptoms of depression. Each item is rated on a 4-point Likert scale ranging from 0 to 3. Total scores range from 0 to 63, with higher scores indicating more severe depressive symptoms. It was developed by Beck and colleagues (15), and a validity and reliability study has been carried out for Turkish society (16).

#### 2.2.3 Behavioral inhibition system/behavioral activation system scale

The Behavioral Inhibition System/Behavioral Activation System (BIS/BAS) Scale was developed by Carver and White (17) and consists of one BIS subscale and three BAS subscales: Drive, Fun Seeking, and Reward Responsiveness (17). The BIS subscale evaluates sensitivity to punishment and avoidance-related behaviors, whereas the BAS subscales assess reward sensitivity, goal-directed behavior, and approach motivation. The scale consists of 24 items rated on a 4-point Likert scale. Higher BIS scores reflect higher sensitivity to punishment and avoidance motivation, whereas higher BAS scores reflect higher sensitivity to reward and approach motivation. Sisman et al. (18) have carried out the Turkish translation and adaptation study. (18).

#### 2.2.4 Diabetes self-management questionnaire

Diabetes self-management behaviors were measured using the 16-item self-reported Diabetes Self-Management Questionnaire (DSMQ), which was designed to investigate the relationship between diabetes self-management and glycaemic control (19). The DSMQ is composed of four subdomains: glucose management, dietary control, physical activity and healthcare utilisation. Items are rated on a 4-point Likert scale, where higher scores indicate better diabetes self-management and treatment adherence behaviors. The Turkish adaptation study has been conducted by Eroglu and Sabuncu (20) with a Cronbach α of 0.85 (20).

#### 2.2.5 Social anhedonia scale

Social anhedonia was evaluated using the Social Anhedonia Scale developed by Mishlove and Chapman (21). The scale measures decreased pleasure and interest in social relationships and interactions. It consists of 40 yes/no items, with higher scores indicating higher levels of social withdrawal and lower levels of enjoyment of social experiences. A Turkish translation and adaptation study has been performed by Cihan et al. (22) with a Cronbach α=0.84 (22).

#### 2.2.6 Physical anhedonia scale

Physical anhedonia was measured using the Physical Anhedonia Scale (23), which assesses the reduced ability to experience pleasure from sensory and physical experiences such as taste, touch, movement, warmth, and sexuality (23). A Turkish translation and adaptation study has been conducted by Baskak et al. (24), with a Cronbach α=0.84 (24). It excluded some items with low item correlations and developed a 50-item yes/no self-report scale. Higher scores indicate greater physical anhedonia.

#### 2.2.7 Monetary choice questionnaire

Delay discounting behavior was assessed using the 27-item Monetary Choice Questionnaire developed by Kirby and colleagues (25). The questionnaire consists of binary choices between smaller immediate monetary rewards and larger delayed monetary rewards. Based on participants’ responses, an overall delay discounting parameter (k) was calculated. Higher k values are associated with a greater preference for immediate rewards and steeper delay discounting, while lower k values are associated with a greater preference for delayed rewards. Because k values were positively skewed, log-transformed k values were used in the statistical analyses.

## 3 Statistical analyses

Statistical analyses were performed using IBM SPSS Statistics version 30.0 for Mac (IBM Corp., Armonk, NY, USA). Normality assumptions were evaluated using Kolmogorov–Smirnov tests, skewness and kurtosis values, and visual inspection of distributions. Group differences in sociodemographic and clinical variables were examined using one-way ANOVA and chi-square tests, as appropriate.

Separate analyses of covariance (ANCOVAs) were conducted to compare reward-related behavioral measures (physical anhedonia, social anhedonia, BIS, BAS Drive, BAS Fun Seeking, BAS Reward Responsiveness, and delay discounting) across groups while controlling for age, sex, education level and depressive symptom severity (BDI scores). Raw means and standard deviations were presented descriptively, whereas statistical comparisons were based on adjusted ANCOVA models. Effect sizes were reported as partial eta squared (partial η²). To control for multiple comparisons, p values from the omnibus ANCOVAs were adjusted using the Benjamini–Hochberg false discovery rate (FDR) procedure. For outcomes showing significant group effects after FDR correction, pairwise group comparisons were performed with Bonferroni adjustment.

Pearson correlation analyses were conducted to assess the associations between reward and motivation-related behavioral measures, diabetes self-management and HbA1c levels in patients. Hierarchical multiple linear regression analyses were subsequently performed to examine whether reward-related behavioral measures explained additional variance in diabetes self-management and HbA1c beyond relevant demographic and clinical covariates. In both analyses, age, sex, depressive symptoms, BMI, and diabetes subtype were entered in the first block. Given the modest sample size and to maintain model parsimony, reward-related behavioral measures showing significant bivariate correlations with the respective outcome were entered together in the subsequent block. Accordingly, BAS Drive, BAS Reward Responsiveness, and social anhedonia were included in the diabetes self-management model, whereas BIS, BAS Drive, and BAS Reward Responsiveness were included in the HbA1c model. For the HbA1c analysis, diabetes self-management was additionally entered in the final block to examine whether the associations with reward-related behavioral measures were retained after accounting for self-management. To evaluate the robustness of the primary HbA1c findings, a sensitivity analysis including all seven reward-related behavioral measures was additionally performed and is presented in the **Supplementary Material**.

Regression assumptions, including linearity, normality and homoscedasticity of residuals, independence of errors, multicollinearity, and influential observations, were examined and considered acceptable. The level of statistical significance was set at p <.05 (two-tailed).

## 4 Results

### 4.1 Characteristics of participants

Sociodemographic and clinical characteristics of the study groups are presented in **Table 1**. Significant group differences were observed regarding age, sex, education level, BMI, HbA1c levels, and depressive symptom severity (all p values ≤.002). Participants with T2DM and prediabetes had higher BMI values compared to healthy controls and individuals with T1DM. The T1DM group had the highest HbA1c levels, followed by the T2DM group. BDI scores were also higher in the prediabetes and T1DM groups compared to healthy controls. No differences in smoking status were observed between groups.

**Table 1:** Sociodemographic and clinical characteristics of study groups.

Variable	T1DM (n=44)	T2DM (n=43)	Pre-diabetes (n=28)	Controls (n=46)	p
Age, mean ± SD	32.66 ± 10.96	52.67 ± 9.58	36.25 ± 12.96	30.15 ± 9.12	*<.001^1^*
Sex(female), n (%)	29 (65.9)	23 (53.5)	27 (96.4)	32 (69.6)	*.002*
Education level, n (%)					*<.001*
Primary school	1 (2.3)	13 (30.2)	1 (3.6)	0	
Elementary school	0	2 (4.7)	5 (17.9)	1 (2.2)	
High school	15 (34.1)	13 (30.2)	13 (46.4)	4 (8.7)	
University	28 (63.6)	15 (34.9)	9 (32.1)	41 (89.1)	
BMI, mean ± SD	25.36 ± 4.72	33.89 ± 5.82	36.47 ± 7.31	23.87 ± 4.18	*<.001^2^*
Smoking (yes), n (%)	15 (34.1)	18 (41.9)	8 (28.6)	14 (30.4)	*.614*
HbA1c, mean ± SD	9.39 ± 1.86	7.72 ± 1.74	5.66 ± 0.33	5.27 ± 0.30	*<.001^3^*
BDI, mean ± SD	13.93 ± 9.30	10.00 ± 7.76	15.32 ± 9.70	8.63 ± 6.48	*.001^4^*

1 T2DM > T1DM =Pre-diabetes=Controls.

2 T2DM =Pre-diabetes> T1DM =Controls.

3 T1DM > T2DM > Pre-diabetes= Controls.

4 T1DM = Pre-diabetes> Controls.

### 4.2 Group comparisons of reward-related behavioral measures

Separate analyses of covariance (ANCOVAs) were performed to compare reward-related behavioral measures among study groups, covarying for age, sex, education level and severity of depressive symptoms.

BIS scores were significantly different between groups (F = 22.53, p <.001, partial η2 = .306) and this effect survived FDR correction. Similarly, BAS Reward Responsiveness scores differed significantly across groups (F = 19.20, p <.001, partial η² = .274), and this finding also survived FDR correction. In contrast, no significant group differences were observed for BAS Drive (FDR-adjusted p = .378), BAS Fun Seeking (FDR-adjusted p = .609), delay discounting (FDR-adjusted p = .266), physical anhedonia (FDR-adjusted p = .661), or social anhedonia (FDR-adjusted p = .661) (**Table 2**).

**Table 2:** Group comparisons of reward-related behavioral measures.

Variable	T1DM (n=44) Mean ± SD	T2DM (n=43) Mean ± SD	Pre-diabetes (n=28) Mean ± SD	Controls (n=46) Mean ± SD	F	P	FDR-adjusted p	Partial η²
BIS	16.43 ± 3.12	20.88 ± 3.25	20.79 ± 3.96	21.26 ± 3.45	22.53	**<.001** **^1^**	**.004**	.306
BAS Reward	14.39 ± 4.36	18.60 ± 1.50	18.61 ± 1.66	18.11 ± 1.69	19.20	**<.001** **^1^**	**.004**	.274
BAS Drive	10.91 ± 3.12	10.21 ± 3.02	11.61 ± 2.95	11.04 ± 2.21	1.73	.162	.378	.033
BAS Fun Seeking	11.64 ± 2.62	10.95 ± 2.81	11.86 ± 2.66	12.35 ± 2.33	1.11	.348	.609	.021
Delay discounting	−1.69 ± 0.59	−1.17 ± 0.66	−1.39 ± 0.89	−1.80 ± 0.64	2.33	.076	.266	.044
Physical anhedonia	14.00 ± 7.95	16.53 ± 7.05	17.07 ± 8.36	12.00 ± 7.40	0.53	.661	.661	.010
Social anhedonia	13.52 ± 5.78	13.16 ± 5.72	14.68 ± 7.13	10.89 ± 6.03	0.77	.510	.661	.015

Raw means and standard deviations are presented for descriptive purposes.

F statistics and p values are based on ANCOVAs adjusted for age, sex, education level and depressive symptoms (BDI).

False discovery rate (FDR) correction was performed using the Benjamini–Hochberg procedure.

Bold values indicate statistically significant associations.

1 T1DM < T2DM, Prediabetes, Controls.

Bonferroni-corrected pairwise comparisons revealed that the T1DM group had significantly lower BIS scores than the T2DM (MD = −5.18, 95% CI [−7.61, −2.76], p <.001), prediabetes (MD = −4.26, 95% CI [−6.50, −2.02], p <.001), and healthy control groups (MD = −5.34, 95% CI [−7.27, −3.40], p <.001). Similarly, BAS Reward Responsiveness scores were significantly lower in the T1DM group than in the T2DM (MD = −3.47, 95% CI [−5.39, −1.55], p <.001), prediabetes (MD = −4.23, 95% CI [−6.00, −2.45], p <.001), and healthy control groups (MD = −3.44, 95% CI [−4.97, −1.90], p <.001). No significant differences were observed among the T2DM, prediabetes, and healthy control groups for either measure (all Bonferroni-adjusted p = 1.000).

### 4.3 Correlations between reward-related behavioral measures, diabetes self-management, and HbA1c

Correlation analyses demonstrated several significant associations between reward-related behavioral measures, diabetes self-management, and glycaemic control (**Table 3**). Higher HbA1c levels were associated with lower BIS scores (r = −.330, p <.001), lower BAS Drive scores (r = −.245, p = .008), and particularly lower BAS Reward Responsiveness scores (r = −.451, p <.001). Delay discounting, physical anhedonia, and social anhedonia were not significantly associated with HbA1c levels.

**Table 3:** Correlations between reward-related behavioral measures, diabetes self-management, and HbA1c in patients (N = 115).

Variable	HbA1c	DSMQ Total
BIS	−.330***	−.059
BAS Drive	−.245**	.280**
BAS Reward	−.451***	.231*
BAS Fun Seeking	−.054	.134
Delay discounting	−.169	.094
Physical anhedonia	−.109	−.183
Social anhedonia	−.066	−.223*

DSMQ, Diabetes Self-Management Questionnaire.

*p <.05, **p <.01, ***p <.001.

Regarding diabetes self-management, higher BAS Drive scores were positively associated with overall DSMQ scores (r = .280, p = .002), whereas social anhedonia was negatively associated with overall DSMQ scores (r = −.223, p = .017). BAS Reward Responsiveness was also positively correlated with overall DSMQ scores (r = .231, p = .013).

Exploratory analyses of DSMQ subdomains demonstrated that higher BAS Drive scores were associated with better dietary control (r = .200, p = .032), increased physical activity (r = .309, p <.001), and greater healthcare utilisation (r = .188, p = .044). Similarly, BAS Reward Responsiveness was positively associated with dietary control (r = .222, p = .017) and healthcare utilisation (r = .383, p <.001). BAS Fun Seeking was positively associated with dietary control (r = .186, p = .046) and physical activity (r = .205, p = .028). Higher BIS scores were associated with lower physical activity (r = −.207, p = .026) and greater healthcare utilisation (r = .273, p = .003). In contrast, social anhedonia was negatively associated with dietary control (r = −.232, p = .012), while physical anhedonia demonstrated a modest negative association with physical activity (r = −.188, p = .044).

### 4.4 Hierarchical regression analyses of diabetes self-management

A hierarchical multiple regression analysis was conducted to examine the incremental contribution of reward-related behavioral measures to diabetes self-management (**Table 4**). Among the reward-related measures, BAS Drive, BAS Reward Responsiveness, and social anhedonia were included because they showed significant bivariate correlations with diabetes self-management. Age, sex, depressive symptoms, BMI, and diabetes subtype were entered as covariates in Model 1, explaining 24.5% of the variance in diabetes self-management (R² = .245, p <.001). The addition of the reward-related behavioral measures in Model 2 explained a further 9.1% of the variance, representing a significant increase in explained variance (ΔR² = .091, p = .004). Within this block, higher BAS Drive was significantly associated with better diabetes self-management (β = .187, p = .040), whereas BAS Reward Responsiveness (β = .209, p = .058) and social anhedonia (β = −.094, p = .283) were not significantly associated with diabetes self-management. The final model explained 33.6% of the variance in diabetes self-management (adjusted R² = .279) and was statistically significant, F(9, 105) = 5.899, p <.001.

**Table 4:** Hierarchical regression analysis of diabetes self-management in patients (N = 115).

Variable	β	t	p	ΔR²
**Model 1**				−
Age	.270	2.457	***.016***	
Sex	.088	0.975	*.332*	
BDI	−.431	−4.899	***<.001***	
BMI	−.247	−2.218	***.029***	
T2DM (ref. T1DM)	−.158	−1.142	*.256*	
Prediabetes (ref. T1DM)	.051	0.421	*.675*	
**Model 2 (Model 1+Reward-related measures)**				.091**
BAS Drive	.187	2.084	***.040***	
BAS Reward Responsiveness	.209	1.920	*.058*	
Social Anhedonia	−.094	−1.080	*.283*	

Final model: R² = .336, Adjusted R² = .279, F(9, 105) = 5.899, p <.001.

Bold values indicate statistically significant associations.

**p <.01.

### 4.5 Hierarchical regression analyses of HbA1c levels

A second hierarchical multiple regression analysis was conducted to examine the incremental contribution of reward-related behavioral measures to HbA1c levels (**Table 5**). Among the reward-related measures, BIS, BAS Drive, and BAS Reward Responsiveness were included in this analysis because they showed significant bivariate correlations with HbA1c. Age, sex, depressive symptoms, BMI, and diabetes subtype were included as covariates in Model 1, which explained 50.7% of the variance in HbA1c (R² = .507, p <.001). In Model 2, the addition of BIS, BAS Drive, and BAS Reward Responsiveness explained a further 3.8% of the variance in HbA1c, representing a significant increase in explained variance (ΔR² = .038, p = .035). Among these measures, lower BAS Drive was significantly associated with higher HbA1c (β = −.168, p = .025), whereas BIS and BAS Reward Responsiveness were not significantly associated with HbA1c after adjustment.

**Table 5:** Hierarchical regression analysis of HbA1c levels in patients (N = 115).

Variable	β	t	p	ΔR²
**Model 1**				−
Age	−.248	−2.802	***.006***	
Sex	−.026	−0.357	*.722*	
BDI	.096	1.351	*.179*	
BMI	−.050	−0.554	*.581*	
T2DM (ref. T1DM)	−.160	−1.432	*.155*	
Prediabetes (ref. T1DM)	−.709	−7.294	***<.001***	
**Model 2 (Model 1+Reward-related measures)**				.038*
BIS	−.043	−0.506	*.614*	
BAS Drive	−.168	−2.271	***.025***	
BAS Reward Responsiveness	−.066	−0.696	*.488*	
**Model 3 (Model 2+ DSMQ)**				.001
BIS	−.043	−0.498	*.619*	
BAS Drive	−.162	−2.124	***.036***	
BAS Reward Responsiveness	−.060	−0.616	*.539*	
DSMQ	−.034	−0.420	*.676*	

Final model: R² = .546, Adjusted R² = .503, F(10, 104) = 12.521, p <.001.

BDI, Beck Depression Inventory; BMI, body mass index; BIS, Behavioral Inhibition System; BAS, Behavioral Activation System; DSMQ, Diabetes Self-Management Questionnaire. T1DM was the reference category for diabetes subtype. Only variables newly entered at each step are displayed.

Bold values indicate statistically significant associations.

*p <.05.

In Model 3, diabetes self-management was additionally included to examine whether these associations were retained after accounting for self-management. The association between BAS Drive and HbA1c remained significant (β = −.162, p = .036), while diabetes self-management itself was not independently associated with HbA1c (β = −.034, p = .676) and explained negligible additional variance (ΔR² = .001, p = .676). The final model explained 54.6% of the variance in HbA1c (adjusted R² = .503) and was statistically significant, F(10, 104) = 12.521, p <.001.

### 4.6 Exploratory analyses

In secondary analyses, BIS, BAS Drive, and BAS Reward Responsiveness were examined separately in models adjusted for age, sex, depressive symptoms, BMI, and diabetes subtype. Lower BAS Drive was associated with higher HbA1c (β = −.191, *p* = .006), and this association remained significant after FDR correction (*q* = .018). Neither BIS (β = −.073, *p* = .385, *q* = .385) nor BAS Reward Responsiveness (β = −.158, *p* = .063, *q* = .095) was significantly associated with HbA1c.

As a sensitivity analysis, a model including all reward-related behavioral measures was examined and is presented in **Supplementary Table S1**. This analysis yielded a pattern broadly consistent with the primary analysis, with BAS Drive emerging as the only reward-related measure significantly associated with HbA1c in the fully adjusted model (β = −.185, p = .027).

## 5 Discussion

The present study investigated motivation and reward-related behavioral measures across different metabolic and insulin states and explored their associations with diabetes self-management and glycaemic control. The main findings were that behavioral inhibition and reward responsiveness differed significantly across groups, whereas anhedonia and delay discounting did not demonstrate robust group differences after correction for multiple comparisons. Among patients, reward-related behavioral measures explained additional variance in both diabetes self-management and HbA1c beyond demographic and clinical covariates, although their contribution to HbA1c was modest. BAS Drive showed the most consistent individual association with HbA1c across the primary and sensitivity analyses. Together, these findings suggest that reward-related behavioral alterations may vary across diabetes subtypes, with a more distinct pattern in T1DM, while specific motivational dimensions may also be relevant to individual differences in glycaemic control. The absence of robust differences in anhedonia and delay discounting further suggests that these findings may reflect specific aspects of reward-related functioning rather than a generalised impairment across reward domains.

One of the most notable findings of the present study was the alteration in BAS-reward responsiveness across metabolic groups. Reward responsiveness reflects sensitivity to rewarding stimuli and positive reinforcement and has been linked to mesolimbic dopaminergic functioning. Experimental studies have demonstrated that insulin signalling modulates dopamine release and dopamine transporter activity within reward-related neural pathways (3, 4). Previous research has shown that diabetes is associated with specific disturbances in reward learning, valuation, and effort, mediated by dopaminergic and salience-related circuits (6, 8, 26, 27). However, relatively little is known regarding potential differences across diabetes subtypes and varying insulin-related conditions. Interestingly, our *post hoc* analyses revealed that individuals with T1DM demonstrated significantly lower BAS-reward responsiveness scores compared to healthy controls, whereas no significant differences were observed between T2DM, prediabetes, and healthy control groups. Given that T1DM is characterised by absolute insulin deficiency requiring exogenous insulin replacement, whereas prediabetes and T2DM are characterised by differing patterns of insulin resistance, compensatory hyperinsulinemia, and relative insulin deficiency, the observed group-specific pattern may be related to differences in insulin-related metabolic physiology. The absence of comparable differences in T2DM and prediabetes may reflect heterogeneity in metabolic physiology, disease characteristics, or treatment exposure across these groups. However, as neither insulin signalling nor dopaminergic function was directly assessed, the underlying mechanisms cannot be determined from the present findings.

Similarly, individuals with T1DM demonstrated significantly lower BIS scores compared to healthy controls. Behavioral inhibition is generally associated with sensitivity to punishment and avoidance-related motivational tendencies. Lower BIS scores in T1DM may reflect altered inhibitory motivational processing and reduced sensitivity to aversive or punishment-related cues. Interestingly, BIS scores were negatively associated with HbA1c levels and physical activity in correlation analyses, suggesting that inhibitory motivational processes may also have behavioral relevance for diabetes management and glycaemic control. However, this association was not retained after adjustment for demographic, clinical, and metabolic factors. Thus, the lower BIS scores observed in T1DM appear to represent a group-level behavioral difference rather than a robust independent correlate of glycaemic control.

Although previous research has linked diabetes and metabolic dysfunction with anhedonia, direct human studies comparing individuals with diabetes and healthy controls using anhedonia measures remain limited. Siwek et al. (28) demonstrated that insulin resistance predicts anhedonia in a transdiagnostic sample (28). In addition, structural and functional alterations across dopamine-based reward circuits, including the striatum, anterior cingulate cortex, and other mesolimbic regions, have been reported in T2DM, suggesting a potential neural basis for motivational and decisional aspects of anhedonia (5). Despite this literature, anhedonia measures did not significantly differ between groups after correction for multiple comparisons in the present study. Similarly, delay discounting did not differ significantly across groups after correction for multiple comparisons and was not associated with HbA1c in the fully adjusted sensitivity analysis. This finding is not fully consistent with the study by Liu et al., which reported greater delay discounting in individuals with T1DM compared to healthy controls (29). However, the literature regarding delay discounting in diabetes is still relatively limited. Taken together, our findings of nonsignificant group differences in anhedonia and delay discounting may suggest that not all reward-related behavioral dimensions are equally affected across insulin-related conditions. Rather than a generalized alteration in reward functioning, the observed pattern may reflect more specific differences in motivational and reward-responsive dimensions. Another consideration is that some reward-related processes may be particularly sensitive to state-dependent influences. Anhedonia, in particular, may include a state-dependent component related to current affective and depressive symptoms rather than exclusively reflecting a stable trait-like alteration. As depressive symptoms were included as a covariate in our adjusted group comparisons, some of the variance in anhedonia associated with current depressive symptom severity may have been statistically accounted for. Previous literature also suggests that difficulties in emotion regulation may be associated with poorer glycaemic control in diabetes, further highlighting the relevance of current affective functioning (30). Beyond affective state, differences in the instruments used to assess reward-related constructs, demographic and clinical characteristics of the samples, metabolic status, disease duration, and treatment exposure may also contribute to variability across studies. These factors may partly explain why some reward-related measures do not show consistent differences across diabetes populations in cross-sectional studies.

An important finding of the present study was that reward-related behavioral measures explained a modest but statistically significant proportion of additional variance in HbA1c after accounting for age, sex, depressive symptoms, BMI, and diabetes subtype. The behavioral block accounted for an additional 3.8% of the variance in HbA1c, indicating that reward-related functioning may contribute to individual differences in glycaemic control beyond these demographic, clinical, and metabolic factors. Among the individual behavioral measures, lower BAS Drive showed the most consistent association with higher HbA1c. Importantly, this association remained significant after additional adjustment for diabetes self-management and was also retained in the sensitivity analysis including all seven reward-related measures. In contrast, the bivariate associations of BIS and BAS Reward Responsiveness with HbA1c were attenuated in the adjusted models. Notably, the behavioral dimension that most clearly differentiated T1DM from the other groups (Reward Responsiveness) was not the same as that most consistently associated with individual variation in HbA1c (Drive). This distinction may indicate that between-group differences in responsiveness to reward and individual variation in goal-directed motivational pursuit capture related but distinct aspects of reward functioning. Previous studies examining the relationship between reward-related processes and glycaemic control have yielded mixed findings. Although anhedonia has been associated with suboptimal glycaemic control in diabetes (31), another study including mixed T1DM and T2DM samples found no significant association between anhedonia scores and HbA1c levels (32). Direct evidence linking BIS/BAS traits with HbA1c in diabetes remains limited. However, in non-diabetic populations, higher BAS scores have been associated with increased fat intake, alcohol consumption, binge drinking, smoking, and smoking frequency, whereas higher BIS scores were associated with increased sugar intake (33). Furthermore, a systematic review using BIS/BAS and related reward-sensitivity measures demonstrated that increased reward sensitivity is consistently associated with binge eating, emotional eating, and increased consumption of palatable foods (34). These eating patterns are well-established risk factors for obesity and T2DM, although direct associations with HbA1c were not examined. Effective diabetes management requires sustained motivation and prioritisation of delayed health benefits over immediate gratification. BAS Drive reflects persistent goal-directed pursuit of desired outcomes, making its association with HbA1c potentially relevant in the context of diabetes, where long-term management requires sustained engagement in behaviors whose benefits may not be immediately reinforcing. Importantly, BAS Drive remained significantly associated with HbA1c after additional adjustment for diabetes self-management, suggesting that this association cannot be fully accounted for by differences in self-reported self-management behaviors. This pattern may also be consistent with broader neurobehavioral mechanisms linking insulin dysregulation with motivational processing. Nevertheless, given the cross-sectional design, the directionality and underlying mechanisms of these associations cannot be determined.

Reward-related behavioral measures were also associated with diabetes self-management, explaining an additional 9.1% of its variance beyond demographic and clinical covariates. Among the individual measures, BAS Drive was significantly associated with better self-management, whereas the association with BAS Reward Responsiveness was of borderline significance. However, diabetes self-management did not explain additional variance in HbA1c in the final model. This finding should be interpreted cautiously, particularly given the well-established clinical relevance of self-management to glycaemic control. Self-reported self-management behaviors may be susceptible to recall and social-desirability biases and may not fully reflect actual self-management practices. Moreover, HbA1c reflects the combined influence of behavioral, biological, disease-related, and treatment-related factors. Thus, the limited incremental contribution of self-reported diabetes self-management to HbA1c in the present model should be considered in the context of both the multifactorial determinants of glycaemic control and the limitations inherent in self-report assessment.

BMI was additionally included in the regression models because of its potential relationship with both metabolic status and reward-related behavior. However, BMI may itself partly reflect behavioral processes related to reward and motivation and may share common determinants with these measures; consequently, adjustment for BMI may also remove some potentially meaningful shared variance.

The present findings should be interpreted in light of several limitations. First, the cross-sectional design precludes causal inferences regarding the relationship between insulin dysregulation, motivation and reward-related behaviors, and glycaemic control. Second, although analyses were adjusted for important covariates including age, sex, BMI, depressive symptoms and diabetes subtypes, the groups differed regarding several sociodemographic and clinical variables, particularly age and sex distribution. Such imbalances may not be fully addressed through statistical adjustment and could have influenced the observed group differences. This is particularly relevant for the prediabetes group, which was predominantly female and should be taken into account when interpreting findings involving this subgroup. Another important limitation is that participants with diabetes were receiving ongoing treatment, including insulin replacement and other antidiabetic medications, which may have confounded the results. Although insulin deficiency has been implicated in altered dopaminergic and reward-related processes, it remains unclear whether peripheral insulin replacement exerts comparable central effects or fully reverses potential central disturbances associated with chronic insulin deficiency. Nevertheless, significant differences were still observed, particularly in the T1DM group, despite ongoing treatment. Accordingly, the observed differences cannot be attributed specifically to insulin deficiency, insulin resistance, or their central neurobiological effects. The selection of behavioral variables for the primary regression models was based on their bivariate associations with the respective outcomes and should therefore be regarded as data-informed and exploratory. However, the sensitivity analysis including all reward-related measures yielded a broadly consistent pattern for HbA1c, with BAS Drive remaining the only reward-related measure significantly associated with HbA1c. In addition, behavioral measures were assessed using self-report questionnaires, which may be influenced by reporting bias and may not fully capture objective reward-related functioning. Finally, the relatively modest sample size may have limited statistical power to detect smaller effect sizes.

Despite these limitations, the present study has several strengths. To our knowledge, this is the first study to simultaneously compare individuals with T1DM, T2DM, prediabetes, and healthy controls regarding motivation and reward-related behavioral functioning. In addition, the study integrated behavioral measures with clinically relevant outcomes, such as diabetes self-management and HbA1c levels. The findings suggest that specific dimensions of reward-related and motivational functioning may represent clinically relevant behavioral correlates of diabetes phenotype, self-management, and glycaemic control.

In conclusion, the present study suggests that alterations in selected dimensions of reward-related behavior may be particularly evident in T1DM, with lower reward responsiveness and behavioral inhibition distinguishing this group from T2DM, prediabetes, and healthy controls. Across patients, reward-related behavioral measures accounted for a modest but significant proportion of additional variance in HbA1c beyond demographic, clinical, and metabolic factors, with BAS Drive showing the most consistent individual association with glycaemic control. Reward-related measures were also associated with diabetes self-management. These findings highlight the potential relevance of motivational and reward-related processes in diabetes. Longitudinal studies incorporating objective behavioral paradigms, neuroendocrine measures, and neuroimaging are needed to clarify the temporal and biological mechanisms underlying these associations.

## Data Availability

The raw data supporting the conclusions of this article will be made available by the authors, without undue reservation.
