# Supplementary material for: Reward-related functioning across diabetes subtypes: associations with glycaemic control
Source: Front Endocrinol (Lausanne). 2026 Sep 18;17:1959733. doi: 10.3389/fendo.2026.1959733 (PMC13630698; doi:10.3389/fendo.2026.1959733)
Supplement: Supplementary file 1 [file Table1.docx]

| ***Supplementary Table S1. Fully Adjusted Regression Analysis Including All Reward-Related Behavioral Measures for HbA1c*** | | | | | | |
| --- | --- | --- | --- | --- | --- | --- |
|  | **B** | **β** | **t** | ***p*** | **95% CI for B** |  |
| Age | −0.036 | −.239 | −2.552 | ***.012*** | −0.064 to −0.008 |  |
| Sex | −0.122 | −.027 | −0.360 | *.720* | −0.793 to 0.549 |  |
| BMI | −0.016 | −.057 | −0.623 | *.535* | −0.068 to 0.036 |  |
| T2DM (ref. T1DM) | −0.530 | −.121 | −0.927 | *.356* | −1.665 to 0.605 |  |
| Prediabetes (ref. T1DM) | −3.124 | −.631 | −5.655 | ***<.001*** | −4.221 to −2.028 |  |
| BDI | 0.014 | .060 | 0.692 | *.490* | −0.027 to 0.055 |  |
| DSMQ Total | −0.034 | −.028 | −0.334 | *.739* | −0.234 to 0.166 |  |
| Delay discounting | −0.069 | −.023 | −0.309 | *.758* | −0.511 to 0.373 |  |
| BIS | −0.017 | −.032 | −0.354 | *.724* | −0.112 to 0.078 |  |
| BAS Drive | −0.129 | −.185 | −2.249 | ***.027*** | −0.242 to −0.015 |  |
| BAS Fun Seeking | 0.045 | .058 | 0.686 | *.494* | −0.086 to 0.177 |  |
| BAS Reward Responsiveness | −0.053 | −.089 | −0.821 | *.414* | −0.180 to 0.075 |  |
| Physical anhedonia | 0.019 | .067 | 0.793 | *.429* | −0.028 to 0.065 |  |
| Social anhedonia | −0.030 | −.087 | −1.037 | *.302* | −0.089 to 0.028 |  |
| *T1DM was the reference category for diabetes subtype.*  ***Bold values indicate statistically significant associations.***  *B = unstandardized regression coefficient; SE = standard error; β = standardized regression coefficient; CI = confidence interval.* | | | | | |  |
